# Is It Time to Screen Women with History of Hypertensive Pregnancy Disorders for Diabetes?

**DOI:** 10.1371/journal.pmed.1001428

**Published:** 2013-04-16

**Authors:** Thach Tran

**Affiliations:** ARCH: Australian Research Centre for Health of Women and Babies, The Robinson Institute, School of Paediatrics and Reproductive Health, The University of Adelaide, North Adelaide, Australia

## Abstract

Thach Tran discusses a new research article on whether preeclampsia is associated with future diabetes, and discusses whether women with a history of hypertensive disorders in pregnancy should be screened for diabetes.

**Linked Research Article**

This Perspective discusses the following new study published in *PLOS Medicine*:

Feig DS, Shah BR, Lipscombe LL, Wu CF, Ray JG, et al. (2013) Preeclampsia as a Risk Factor for Diabetes: A Population-Based Cohort Study. PLoS Med 10(4): e1001425. doi:10.1371/journal.pmed.1001425

Denice Feig and colleagues assess the association between gestational diabetes, gestational hypertension, and preeclampsia and the development of future diabetes in a database analysis of pregnant women in Ontario, Canada.

Diabetes mellitus (DM) is a growing problem, with an estimated global prevalence of 9.9% or 552 million adults affected in 2030 [1]. Evidence from randomised controlled trials has proved that several interventions, such as lifestyle changes or anti-diabetic medications, can significantly delay or prevent the occurrence of DM in women with impaired glucose intolerance [2]. Identification of risk factors for developing DM is therefore crucial in daily practice since screening high-risk populations for DM and impaired glucose tolerance, with appropriate interventions for those with impaired glucose tolerance, has been deemed cost effective [3]. A Canadian cohort study in this week's *PLOS Medicine* [4] provides valuable new evidence on preeclampsia (PEC) and the impetus for discussing whether it is now time to consider screening women with a history of hypertensive pregnancy disorders.

Gestational diabetes (GDM) is known as a strong risk factor for subsequent diabetes [5], and women with GDM have been consistently recommended to undergo postpartum screening for DM [6],[7]. Like GDM, hypertensive pregnancy disorders have been found to be associated with insulin resistance, which plays an important role in the aetiology of type 2 DM [8], making the group of women with a history of hypertensive disorders in pregnancy a potential candidate for DM prevention programs. Hypertension is more commonly identified than GDM in pregnancy [9]. In a recent international comparative report [9], hypertension was noted in 9% of pregnancies in Australia, 7% in the United States, and 4% in Sweden, compared with GDM in 4.8%, 4.4%, and 0.9% of pregnancies, respectively.

It has been shown that hypertensive disorders in pregnancy, especially PEC, are independently associated with an elevated risk of women subsequently developing DM [10]–[12]. However, the presence of GDM was not taken into account in these previous studies, making clinical interpretation difficult. The new study in *PLOS Medicine*, a population-based retrospective cohort study by Denise Feig and colleagues [4], helps overcome this challenge by examining whether hypertensive pregnancy disorders, including both gestational hypertension (GH) and PEC, increase the risk of subsequent development of DM in the absence of GDM, and how the risks would be changed in the presence of GDM.

## Hypertensive Pregnancy Disorders Alone Doubled the Risk of Developing Diabetes

In Feig and colleagues' study, the cohort of one million women aged 15 to 50 years who delivered in Ontario, Canada, was followed with a median follow-up of 8.5 years [4]. Hypertensive disorders in pregnancy were categorised into four mutually exclusive groups: GE alone, PE alone, GE and GDM, and PEC and GDM, using the hospitalization records and outpatient data from physicians' services claims. Diabetes was determined using the Ontario Diabetes Database, which had been well validated against primary health care charts. A Cox proportional regression was carried out to make an adjustment for potential confounding effects, including maternal age, prior hypertension, socioeconomic status, parity, and co-morbidity. Hypertensive disorders in pregnancy were found to double the risk of developing DM until 16 years after delivery in the absence of GDM, but to confer an almost 20-fold increased risk in the presence of GDM. Approximately 25% of women with GDM and hypertensive disorders are reported to have developed diabetes within the first 5 years following pregnancy, compared with 19% of women with only GDM.

The study is the largest population-based report with successful and long-term follow-up, and is the first to examine the association between hypertensive disorders during pregnancy and subsequent development of diabetes, taking GDM into consideration. As a result, its findings have important clinical and public health implications. However, there are several limitations inherently associated with the retrospective nature of the study. Most importantly, some known risk factors for the development of DM—such as obesity, family history, physical activity, and glucose and blood pressure measures—were not available in the databases, and therefore could not be adjusted for. It is unlikely that this limitation would alter the findings significantly [10].

## Should Women with History of Hypertensive Pregnancy Disorders Be Systematically Screened for Diabetes?

Given current trends in the prevalence of DM and hypertension in pregnancy, and their associated risk, it is time to consider an appropriate action for women with a history of hypertensive pregnancy disorders. Currently, women with a history of hypertensive disorders in pregnancy would not be systematically screened for diabetes for the following reasons.

Firstly, postpartum screening for DM, which often focuses only on women with GDM, has been sub-optimally implemented [13], although it has been consistently recommended for decades [6],[7]. In Tovar et al.'s study, only about half of eligible women in most populations, varying from 34% to 73%, were found to undergo any types of screening postpartum, including fasting plasma glucose and oral glucose tolerance tests [13]. Even in a setting with a high overall screening rate of 73%, only 27% of women were screened accordingly to the current guidelines [14]. Some barriers to postpartum screening DM might not be solved soon [13].

Secondly, there is currently a lack of evidence of benefits of screening women with hypertensive disorders in pregnancy for DM. In fact, a policy for screening the population of women at high risk for DM but not impaired glucose intolerance, with no intervention offered to those with impaired glucose tolerance, was associated with uncertain cost effectiveness [4].

## How Should Women with History of Hypertensive Disorders in Pregnancy Be Managed?

While the current evidence base does not support the idea that women with a history of hypertensive disorders in pregnancy be systematically screened for DM, inaction is not an option either. Based upon this new study, women experiencing hypertensive pregnancy disorders with or without GDM should be considered as a population at high risk for subsequently developing diabetes. All women with a history of hypertensive disorders in pregnancy should thus be counselled about their potential increased risk of subsequent DM and the possible opportunity for screening as well as preventive interventions. Postpartum screening for DM should be individualised. Current postpartum screening programs should particularly focus on women with GDM in combination with hypertensive disorders. Given their higher risk of developing diabetes, it is crucial to exercise more efforts to improve compliance of this high-risk population to the current postpartum screening for diabetes.

## Abbreviations

DM: diabetes mellitus
GH: gestational hypertension
GDM: gestational diabetes
PEC: preeclampsia
